# Genome-Wide Identification and Expression Analysis of Metal Tolerance Protein Gene Family in *Medicago truncatula* Under a Broad Range of Heavy Metal Stress

**DOI:** 10.3389/fgene.2021.713224

**Published:** 2021-09-07

**Authors:** Ahmed H. El-Sappah, Rania G. Elbaiomy, Ahmed S. Elrys, Yu Wang, Yumin Zhu, Qiulan Huang, Kuan Yan, Zhao Xianming, Manzar Abbas, Khaled A. El-Tarabily, Jia Li

**Affiliations:** ^1^School of Agriculture, Forestry and Food Engineering, Yibin University, Yibin, China; ^2^Genetics Department, Faculty of Agriculture, Zagazig University, Zagazig, Egypt; ^3^Faculty of Pharmacy, Ahram Canadian University, Cairo, Egypt; ^4^Soil Science Department, Faculty of Agriculture, Zagazig University, Zagazig, Egypt; ^5^College of Tea Science, Yibin University, Yibin, China; ^6^Department of Biology, College of Science, United Arab Emirates University, Al Ain, United Arab Emirates; ^7^Harry Butler Institute, Murdoch University, Murdoch, WA, Australia

**Keywords:** *Medicago truncatula*, heavy metals, metal tolerance protein (MTP), genome-wide identification, gene expression

## Abstract

Metal tolerance proteins (MTPs) encompass plant membrane divalent cation transporters to specifically participate in heavy metal stress resistance and mineral acquisition. However, the molecular behaviors and biological functions of this family in *Medicago truncatula* are scarcely known. A total of 12 potential *MTP* candidate genes in the *M. truncatula* genome were successfully identified and analyzed for a phylogenetic relationship, chromosomal distributions, gene structures, docking analysis, gene ontology, and previous gene expression. *M. truncatula MTPs* (*MtMTPs*) were further classified into three major cation diffusion facilitator (CDFs) groups: Mn-CDFs, Zn-CDFs, and Fe/Zn-CDFs. The structural analysis of *MtMTPs* displayed high gene similarity within the same group where all of them have cation_efflux domain or ZT_dimer. *Cis*-acting element analysis suggested that various abiotic stresses and phytohormones could induce the most *MtMTP* gene transcripts. Among all *MTPs*, PF16916 is the specific domain, whereas GLY, ILE, LEU, MET, ALA, SER, THR, VAL, ASN, and PHE amino acids were predicted to be the binding residues in the ligand-binding site of all these proteins. RNA-seq and gene ontology analysis revealed the significant role of *MTP* genes in the growth and development of *M. truncatula*. *MtMTP* genes displayed differential responses in plant leaves, stems, and roots under five divalent heavy metals (Cd^2+^, Co^2+^, Mn^2+^, Zn^2+^, and Fe^2+^). Ten, seven, and nine *MtMTPs* responded to at least one metal ion treatment in the leaves, stems, and roots, respectively. Additionally, *MtMTP1.1*, *MtMTP1.2*, and *MtMTP4* exhibited the highest expression responses in most heavy metal treatments. Our results presented a standpoint on the evolution of *MTPs* in *M. truncatula*. Overall, our study provides a novel insight into the evolution of the *MTP* gene family in *M. truncatula* and paves the way for additional functional characterization of this gene family.

## Introduction

Metals act as a cofactor, which has essential implications in inactivating enzymes in plant cells to perform specific biological reactions (Thomine and Vert, 2013). Essential metals including zinc (Zn), manganese (Mn), iron (Fe), cobalt (Co), and copper (Cu), play an essential role in plants at a low level, but excessive amounts of these ions lead to toxic effects (Kolaj-Robin et al., 2015). Moreover, a deficient concentration of non-essential metals, including mercury (Hg), silver (S), cadmium (Cd), and lead (Pb), can also cause plant cell toxicity (Clemens, 2001). Interestingly, plants are natural bio-accumulators for various heavy metals from the water and soil for appropriate plant growth and development activities (Ali et al., 2013; Elrys et al., 2018).

Plants overcome heavy metal stress by various physiological and molecular mechanisms, including genomic-level and complex biochemical processes (Liu et al., 2019). Some of these mechanisms are part of the homeostatic process and are constitutive (Rai et al., 2019). Other mechanisms are exclusively related to counter-specific metal toxicity (Gupta et al., 2019). All responses can be widely classified as being tolerant or avoidance types (Krzesłowska, 2011). Metal uptake, trafficking, storage, chelation, and efflux are plant mechanisms to maintain metal homeostasis (Montanini et al., 2007). Numerous studies have indicated the essential roles of various protein families with their specific transporters in these regulatory processes (Gao et al., 2020). The cation diffusion facilitator (CDF) family genes are integral membrane divalent cation transporters involved in divalent metal ion efflux from the cytoplasm either into subcellular compartments or outside the cell (Gustin et al., 2011). CDF transporters have been widely identified in many organisms since their first identification in the bacterial cell (Nies and Silver, 1995), which were further classified into three major groups: Mn-CDF, Zn/Fe-CDF, and Zn-CDF, based on either confirmed or hypothesized transported substrate specificities (Montanini et al., 2007).

Cation diffusion facilitator transporters are considered as metal tolerance proteins (MTPs) in plants, which were classified into seven distinct groups (1, 5, 6, 7, 8, 9, and 12) based on the annotation and phylogenetic analysis in *Arabidopsis* (Gustin et al., 2011). Many MTP proteins were identified in several plant species, including *Arabidopsis thaliana* (van der Zaal et al., 1999), *Vitis vinifera* (Shirazi et al., 2019), *Populus trichocarpa* (Gao et al., 2020), *Triticum aestivum* (Vatansever et al., 2017), *Nicotiana tabacum*, *Nicotiana sylvestris*, and *Nicotiana tomentosiformis* (Liu et al., 2019). Many Zn-CDF proteins have been studied from the first identified *AtMTP1* in *Arabidopsis* (van der Zaal et al., 1999). Zn-CDF genes play an essential role in plant Zn tolerance. For instance, *AtMTP1* and *AtMTP3* of the tonoplast are involved in Zn and Co tolerance through the excess transport of Zn^2+^ and Co^2+^ ions to the vacuole (Dräger et al., 2004; Kobae et al., 2004; Arrivault et al., 2006; Kawachi et al., 2008). Moreover, two more genes of Zn-CDF family, including *AtMTP5* and *AtMTP12*, were identified to form a functional complex during Zn transportation into the Golgi apparatus (Fujiwara et al., 2015).

Mn-CDF family members, including *AtMTP8*, play an essential role in the transportation of Mn besides its role in Fe and Mn localization in seeds (Delhaize et al., 2007; Eroglu et al., 2016; Chu et al., 2017). In *Oryza sativa*, the *OsMTPs* (*OsMTPs8.1* and *8.2*) of the tonoplast participate in Mn transport within the plant (Takemoto et al., 2017; Tsunemitsu et al., 2018). Moreover, the *ShMTP* gene in *Stylosanthes hamata* was the first group 8 member of *MTPs* to be identified that enhances the tolerance against Mn when overexpressed in *Arabidopsis* (Delhaize et al., 2007). Furthermore, the *CsMTP8* gene of cucumber confers Mn tolerance when overexpressed in yeast and *Arabidopsis* (Migocka et al., 2014).

*Medicago truncatula* is a diploid plant species (2*n* = 2*x* = 16) which is comparatively small, and its genome has been sequenced which is being recursively used as a model plant for legume genetic research (Benedito et al., 2008; Young et al., 2011; Tang et al., 2014). It is one of the best essential forage crops widely cultivated across the world, the same as alfalfa (Young and Udvardi, 2009). *M. truncatula* is the best species to explore functional genomics of metal tolerance, although it does not accumulate metals by itself (Zhou et al., 2012).

However, only a few *M. truncatula MTPs* (*MtMTPs*) have been studied and characterized. In recent years, genome sequencing of model plants and commercially important plants were performed and provided opportunities to screen candidate genes (Edwards et al., 2017). Complete high-quality draft genomes provided a chance to systematically analyze the *M. truncatula MTP* gene family at the genome-wide level.

In the present study, 12 *MTPs* in *M. truncatula* were successfully identified and characterized based on their structural, functional, and evolutionary relationship. Furthermore, change in their expression level was evaluated under the stress of the following five heavy metals: Cd^2+^, Co^2+^, Mn^2+^, Zn^2+^, and Fe^2+^. Our findings will provide a deep insight into the *MTP* gene family involved in heavy metal stress response in a plant cell, founders, and biological functions of MtMTP proteins that will open new avenues of research in the area of the molecular mechanism of homeostasis and heavy metal transport and finally will help to precisely engineer *M. truncatula* plants for heavy metal stress.

## Materials and Methods

### Identification of *MTP* Genes in *M. truncatula*

The *M. truncatula* genome sequence was 2 from an online available *M. truncatula* genome database^1^, and a local database was constructed using BioEdit 7.0 software. Candidate *MtMTP* genes were analyzed for HMM profiling of the following two *MTP* domains PF16916 and PF01545 on the Pfam website^2^. The blast was investigated with our putative MTP protein sequences against the *M. truncatula* whole-genome sequence on NCBI^3^ and Phytozome^4^. All retrieved MTP protein sequences were analyzed at an *E*-value of <10^–5^ to identify the MTP domain using SMART^5^ tools (Letunic et al., 2004). All detailed genetic information of the putative *MTP* gene family, including chromosomal location and CDS, was obtained from the Phytozome database (see text footnote 4). Furthermore, MTP family proteins were analyzed for their molecular weight, number of atoms, amino acids, isoelectric point (PI), molecular weight, and instability index using ExPASy ProtoParam^6^ (Gasteiger et al., 2003). Finally, the subcellular localization data were predicted via the MTP amino acid sequences using protein subcellular localization prediction tools^7^.

### Phylogenetic Analysis

In addition to *M. truncatula*, *MTP* gene family members of *A. thaliana*^7^, *Cucumis sativus*^8^, *P. trichocarpa*^9^, *O. sativa*^10^, and *T. aestivum*^11^ were also retrieved and analyzed for the phylogenetic tree of evolutionary *MTP* relationships. CLUSTALX version 2.0 with default parameters were used for MTP protein multiple sequence alignments. All alignments were uploaded in the MEGA 6.0.6 software with a neighbor-joining method to construct a phylogenetic tree. Finally, bootstrap analysis was performed for 1,000 iterations using a pair-wise gap deletion mode (Tamura et al., 2011).

### Chromosomal Locations and Synteny Analysis

The *M. truncatula* genetic database (see text footnote 4) has been analyzed to retrieve information about chromosomal localization of *MTP* genes, which were subsequently used to construct a genetic map using MapChart software^12^. Two genes of the same species located in the same clade were defined as co-paralogs to identify tandem or segmental duplication events. Simultaneously, the Phytozome database (see text footnote 4) has been analyzed to identify segmental duplication in *M. truncatula* genes. The paralogs were identified, which were considered the results of tandem duplication due to the splicing of two genes into five or more genes within a 100 kb region (Tang et al., 2008). Similarly, co-paralogs located within duplicated chromosomal regions were considered segmental duplications (Wei et al., 2007). The Smith–Waterman algorithm^13^ was used to calculate local alignments of two protein sequences. The synteny analysis of the *MtMTP* gene family was performed using Circos^14^ to localize different alleles distributed on different chromosomes (Krzywinski et al., 2009).

### Gene Structures, Motif Analyses, and *Cis*-Regulatory Element Prediction

A structural analysis of all *MTP* gene family members was performed to explore the organization of intron/exon using both gDNA and CDS sequences by deploying an online tool, namely, Genes Structure Display Server program^15^ (Hu et al., 2015). The conserved gene family motifs were detected using Multiple EM for Motif Elicitation^16^ by adjusting the following parameters: a maximum of 20 motifs and 6–200 amino acids per motif (Bailey et al., 2006). The *cis*-acting elements (CREs) of the *MtMTP* promoter were identified using the PLACE^17^ and PlantCARE programs^18^ (Higo et al., 1999; Lescot et al., 2002).

### Protein Modeling, Prediction, Docking Analysis of the Pocket Sites, and Gene Ontology Annotation (GO)

The Phyre2^19^ website was used for protein modeling, prediction, and analysis of MtMTP proteins (Kelley et al., 2015). The predicted protein model validation was assessed using Ramachandran plot analysis. Docking analysis of the ligand-binding regions in the predicted protein models was also performed using the deep site (Jiménez et al., 2017) and Proteins *Plus*^20^ online analysis software DoGSiteScorer (Volkamer et al., 2012) tools, and was finally visualized in PyMOL^21^.

Blast2GO v3.0.11^22^ and OmicsBox software were applied on all identified MTP protein sequences for GO annotation analysis (Conesa and Götz, 2008).

### Gene Expression Analysis Based on RNA-seq Data

RNA-seq data obtained from different organs of *M. truncatula* was downloaded from the gene Expression Atlas Project (MtGEA)^23^ (He et al., 2009). Furthermore, the retrieved data were analyzed for the actual expression level of *MTP* genes obtained from the leaves, roots, seed coats, and flowers of *M. truncatula* under normal conditions. Subsequently, *MTP* gene expression was also analyzed by deploying cufflinks (version: 2.2.1). Finally, absolute FPKM values were divided by their mean and were transformed into a ratio of log_2_, and MeV 4.5 was used to cluster expression data into a heat map^24^ (Saeed et al., 2006; Babicki et al., 2016).

### Growth Conditions and Heavy Metal Treatments

In this study, an *M. truncatula* (cv. JemalongA17) line was cultivated during the autumn of 2020 at the experimental greenhouse of Yibin University (China). First, the seeds were washed with 10% hypochlorous acid and distilled water. The seeds were germinated using water-saturated filter paper and then transferred to fertilized pittmoss soil with germination conditions of 16-h light (27°C) and 8-h dark (18°C) with relative humidity of 70%. Four seeds were planted in each plastic pot. After emergence, thinning was performed to maintain two uniform seedlings per pot. Thirty-day-old seedlings of *M. truncatula* were placed in 1/2 Hoagland solutions (pH 6.0) with different heavy metal concentrations of 0.1 mM of CdCl_2_, 0.1 mM of CoCl_2_, 0.5 mM of FeSO_4_, 1 mM of MnSO_4_, and 0.5 mM of ZnSO_4_, respectively, and in normal 1/2 Hoagland solutions as the control (CK) (Desoky et al., 2020; Gao et al., 2020). The experimental pots were positioned in a completely randomized block design. The experiment comprised six treatments, as shown above, and each treatment was repeated with three pots. Then, 24 h later, the leaves and roots of tube plantlets were collected and were used as RNA extraction materials. Three biological replicates of the expression analyses were performed for each treatment.

### RNA Extraction and Quantitative Reverse Transcription (qRT)-Polymerase Chain Reaction (PCR) Analysis

TRIzol reagent (Invitrogen, United States) was used for RNA extraction from all plant samples (leaf, stem, and root) and subsequently reverse-transcribed to cDNA using a SuperMix Kit (Transgen, Beijing). The Primer 5.0 tool was used to design specific primers of all selected *MtMTP* genes, including β-actin as a housekeeping gene (Supplementary Table 1). In total, 20 μL of reaction mixture was used to perform real-time polymerase chain reaction (PCR) containing the following reagents: 10 μL of 2 × SYBR premix Taq, 1 μL of cDNA, 0.5 μL of each primer, and 8 μL of ddH_2_O (El-Sappah et al., 2017). Real-time PCR reaction conditions were adjusted as follows: 95°C for 10 min, 95°C for 15 s, 60°C for 60 s, and 40 cycles in total. The relative expression level was calculated by applying the Livak equation 2^–ΔΔCT^ with three replications for each sample (Livak and Schmittgen, 2001).

### Statistical Analysis

Three biological replicates of expression analyses were performed using ± standard deviation at *p* < 0.05. The significant variations between means were compared at *p* < 0.05 using Student’s *t*-test.

## Results

### Identification of *MTP* Genes in *M. truncatula*

In total, 27 genes have been identified using blast analysis; subsequently, genes with an incomplete functional domain were excluded for the next study, and finally, 12 candidate genes were selected for further analysis. Every gene was assigned with a specific name, i.e., *MtMTP1.1*, *MtMTP1.2*, *MtMTP2*, *MtMTP4*, *MtMTP5*, *MtMTP7*, *MtMTP8.1*, *MtMTP8.2*, *MtMTP9*, *MtMTP10.1*, *MtMTP10.2*, and *MtMTP11*. The characteristics of the MtMTP genes were analyzed in detail (Table 1). The length of the CDS sequence of MtMTP genes ranged from 1,158 bp (*MtMTP1.2*) to 1,476 bp (*MtMTP2*), whereas the length of their encoded protein ranged from 347 to 491 amino acids, and the relative MW ranged from 39,491.59 to 53,259.81 Da. Most of the MtMTP proteins have relatively low isoelectric points (pI < 7), except for *MtMTP10.2* and *MtMTP5*, which have a pI of 7.16 and 7.76, respectively. The GRAVY of the MtMTPs ranged from −0.109 (*MtMTP10.2*) to 0.201 (*MtMTP1.2*). The total number of inter- and intra-protein ionic residues were variable, i.e., the highest anionic residues were in *MtMTP1.1* and lowest in *MtMTP5*. Similarly, the highest cationic residues were in *MtMTP10.2* and the lowest in *MtMTP4* and *MtMTP11*. All MtMTP members harbored a variable number of introns, but *MtMTP1.1*, *MtMTP1.2*, and *MtMTP4* did not have any introns. Subcellular localization prediction results showed that all MtMTP proteins were localized to the vacuole membrane (tonoplast), except for *MtMTP2* in the chloroplast, *MtMTP4* in the endoplasmic reticulum, and *MtMTP10.2* in cytoplasm.

**TABLE 1:** The characteristics of *MTP* genes in *Medicago truncatula*.

MTP	Gene NCBI symbol	Location	(−)	(+)	CDS Length (bp)	MW (Da)	aa	Instability	Aliphatic index	GRAVY	PI	Subcellular localization
*MtMTP1.1*	LOC11443599	Chro2; 15887267.15891212	53	29	1224	45054.52	407	31.92	106.81	0.053	5.89	Tonoplast
*MtMTP1.2*	LOC25500609	Chro 8; (8867176.8870206	45	30	1158	42458.02	385	31.15	112.7	0.201	6.02	Tonoplast
*MtMTP2*	LOC25484765	Chro 1; 43633283.43640692	48	39	1476	53259.81	491	40.20	89.06	–0.097	6.43	Chloroplast
*MtMTP4*	LOC25492544	Chro 4; 32388504.32391373	36	26	1185	43976.56	394	27.69	100.91	0.080	6.33	Endoplasmic reticulum
*MtMTP5*	LOC11428206	Chro 7; 43924325.43932959	33	34	1173	43507.07	390	42.01	97.21	0.177	7.76	Tonoplast
*MtMTP7*	LOC25491241	Chro 4; 1626723.1631936	42	40	1317	48487.48	438	36.64	92.65	0.028	6.83	Tonoplast
*MtMTP8.1*	LOC11413755	Chro 3; 31742366.31745903	50	38	1212	45251.13	403	49	109.83	0.062	5.3	Tonoplast
*MtMTP8.2*	LOC11425928	Chro 5; 33499438.33502150	46	30	1188	44510.32	395	38.79	102.91	0.115	5.22	Tonoplast
*MtMTP9*	LOC25501161	Chro 8; 18810864.18814971	45	41	1185	44922.87	394	47.54	96.22	–0.091	6.53	Tonoplast
*MtMTP10.1*	LOC25486917	Chro 2; 33136428.33141227	46	44	1182	45006.21	393	43.79	95.98	–0.015	6.62	Tonoplast
*MtMTP10.2*	LOC11432698	Chro 3; 39685592.39688357	47	47	1206	46241.68	401	40.83	100.4	–0.109	7.16	Cytoplasm
*MtMTP11*	LOC11438849	Chro 7;7882546.7888430	41	26	1203	39491.59	347	44.73	102.59	0.148	5.07	Tonoplast

*(−), (+), MW, aa, GRAVY, and PI denote the total number of negatively charged residues (Asp + Glu), total number of positively charged residues (Arg + Lys), molecular weight, amino acid number, grand average of hydropathicity, and isoelectric points, respectively.*

### Phylogenetic Analysis of *MTP* Gene Families

**FIGURE 1:**
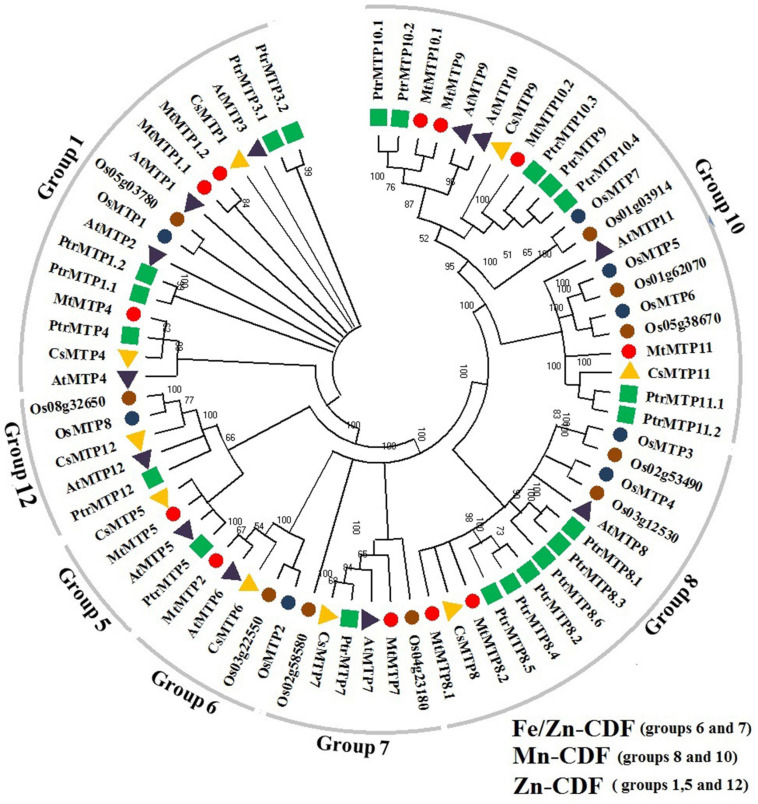
Phylogenetic tree of 72 MTP proteins: 12 *M. truncatula* (marked by red circle), 12 *Arabidopsis* (purple triangle), 8 wheat (blue circle), 10 rice (brown circle), 9 cucumber (yellow triangle), and 21 black poplar (blue square). ClustalX1.83 was used for protein alignments and the phylogenetic tree’s construction at the neighbor-joining (NJ) level using MEGA6.0.6 software at 1,000 replications bootstrap.

All *MTP* gene families were divided into seven groups, i.e., groups 1, 5, 6, 7, 8, 10, and 12 (Figure 1).

The highest number of *MTPs* were pooled in group 10 including *MtMTP9*, *MtMTP10.1*, *MtMTP10.2*, and *MtMTP11* along with *AtMTP9*, *AtMTP10*, and *AtMTP11*; then in group 1 including *MtMTP1.1*, *MtMTP1.2*, and *MtMTP4* along with *AtMTP1*, *AtMTP2*, *AtMTP3*, and *AtMTP4*; then in group 8 including *MtMTP8.1* and *MtMTP8.2* along with *AtMTP8*; then in group 7 including *MtMTP7*, along with *AtMTP7*; then in group 6 including *MtMTP2*, along with *AtMTP6*; and finally in group 5 including *MtMTP5*, along with *AtMTP5*, and no *MtMTPs* were placed in group 12. Noticeably, ionic clustering has revealed that four, two, and six *MtMTP*s were clustered in the Zn-CDFs, Fe/Zn-CDFs, and Mn-CDFs groups, respectively (Figure 1).

### Chromosomal Locations and Synteny Analysis of the *MtMTP* Gene Family

**FIGURE 2:**
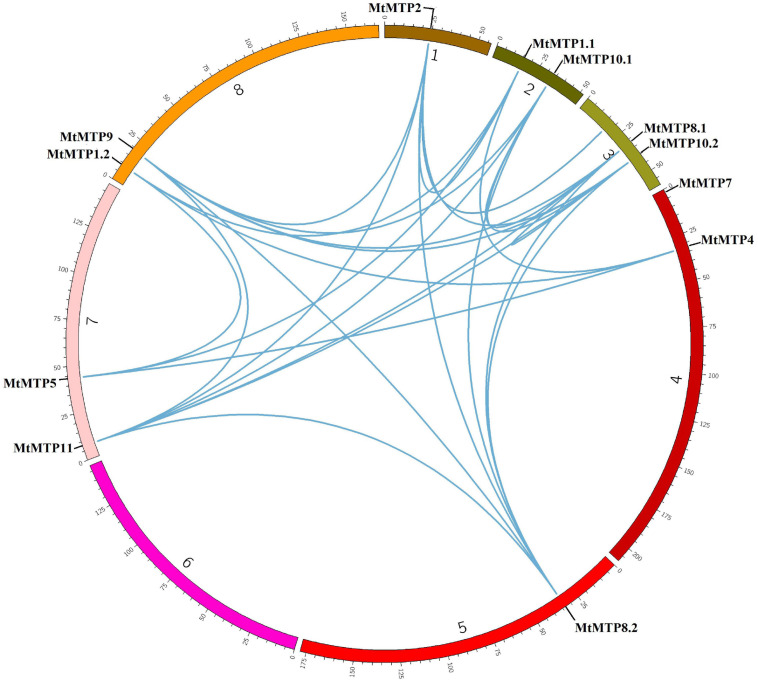
Genome-wide synteny analysis of *MTP* gene family among eight *M. truncatula* chromosomes. The blue lines represented the syntenic orthologs and paralogs and displayed segmental duplication.

Synteny analyses were performed to unravel the distribution of genes on different chromosomes. *MtMTP* genes have been observed to be distributed among all seven chromosomes. Only segmental gene pair duplication has been observed from the Plant Genome Duplication Database. Collinearity due to excision of segmental duplication was observed in many gene pairs with a 70–100% identity percentage (Supplementary Table 2). Segment duplication resulted in many homologies of *MTP* genes between *M. truncatula* chromosome pairs, including those that occur with the genes, *MtMTP1.1*/*MtMTP1.2*, *MtMTP4*/*MtMTP5*, and *MtMTP8.2*/*MtMTP10.2* (Figure 2). Except for *MtMTP7*, all the rest of the *MTP* genes in *M. truncatula* displayed single and multiple genetic duplications. Noticeably, any obvious tandem duplication was not observed among all *MtMTPs*.

### Gene Structures, Motif, and *Cis*-Acting Element Analyses

**FIGURE 3:**
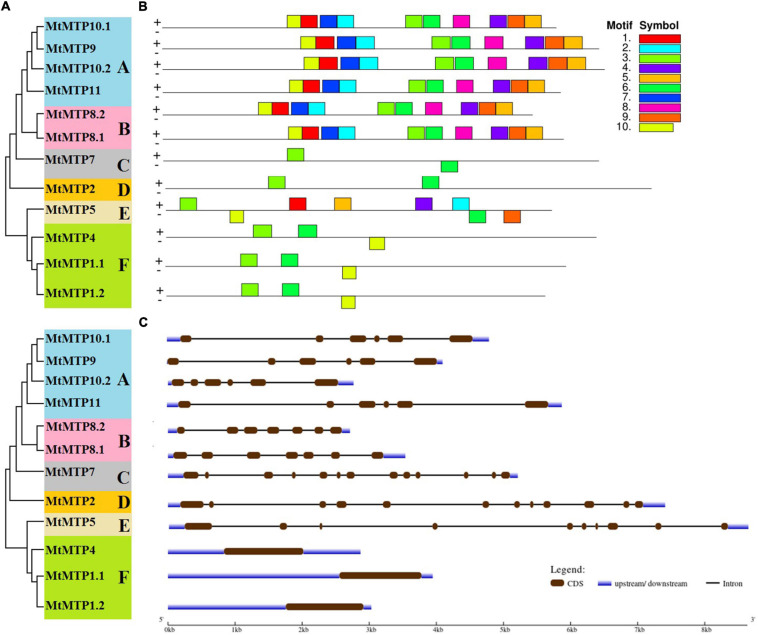
Phylogenetic relationship, gene structure, and conserved motif analysis of *MtMTP* genes. **(A)** The neighbor-joining phylogenetic tree was constructed using MEGA6.0.6 using MtMTP amino acid sequences with 1,000 times replication. **(B)** The motif composition of MtMTP proteins using 10 conserved motifs is represented by the unique color mentioned in the box on the top left. **(C)** The exon-intron structure of *M. truncatula* MTP proteins, where dark green boxes represent the exons, and the black lines represent the introns. The blue boxes represent the untranslated regions (UTRs), with size scales detailed at the bottom.

All *M. truncatula MTP* family genes were further divided into six subfamilies (A, B, C, D, E, and F) (Figure 3A). Subfamilies A and F were the largest among all subfamilies with six members each, followed by subfamily B with two members, whereas subfamilies C, D, and E contained only one gene each (Figure 3A). Intron and exon analysis of all *MTP* genes revealed that each retrieved sequence of the *MtMTP* gene family is a correct and true member of the six subfamilies (Figure 3C). Although the size and location of the intron and exon of all *MtMTP* genes varied, the similarity index was higher among all subfamilies, which proved a close evolutionary relationship among *MtMTP* gene family members. All *MTP* family genes contain a variable number of introns, but all members of subfamily F did not have any introns. Amino acid sequence-based conserved motifs of *MTP* were analyzed using Multiple EM for Motif Elicitation (Figure 3B and Supplementary Table 3). All conserved motifs comprised 50 amino acids, except for motif 10 which comprised only 41 amino acids. The largest motifs were 3 and 6, which were observed in all subfamilies, followed by motif 10, which was 83.3% of the aforementioned motif. Noticeably, the number, type, and order of motifs were more similar in the intrasubfamily than in the intersubfamily.

**FIGURE 4:**
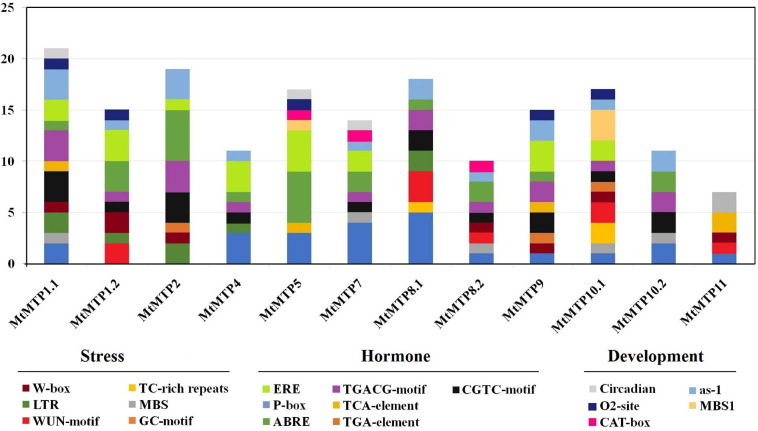
*Cis*-regulatory elements in the promoter region of *MtMTP* genes. *Cis*-regulatory elements were identified in 2,000 bp promoter sequences upstream of the start codon of *MtMTP* genes using the online tools PlantCARE and PLACE. These elements are related to different functional diversity, represented by different colors.

*Cis*-elements related to different stress responses (ARE, WUN-motif, LTR, and MBS) were also found in the promoters of most *MtMTPs* (Figure 4). Furthermore, *cis*-elements involved in plant development (MBS1, CAT-box, ERE, O_2_^–^ site, and EBRE) were found in the promoter region of almost all *MtMTPs*. Meanwhile, the *cis*-elements of the whole *MtMTP* family were divided into different categories based on function prediction. The result showed that the largest category of *cis*-elements was hormone-related, followed by stress-related and developmental-related *cis*-elements. However, many motifs have not yet been functionally characterized, and whether these motifs confer unique functional roles to *MtMTPs* remains to be further investigated.

### Protein Modeling and Docking Analysis of Ligand-Binding Regions

The protein structures of all the candidate MTP proteins were modeled at >90% confidence using the Phyre 2 web portal^19^ (Supplementary Figure 1 and Supplementary Table 4), and their potential active ligand-binding sites were also identified. According to the protein structure results, different active ligand-binding sites were predicted to be MTP proteins (Supplementary Figure 2). Some diversity in the protein structure may reflect their various roles in the transmembrane transport process in response to multiple environments. Additionally, the binding region/active sites of MTP proteins were predicted.

**FIGURE 5:**
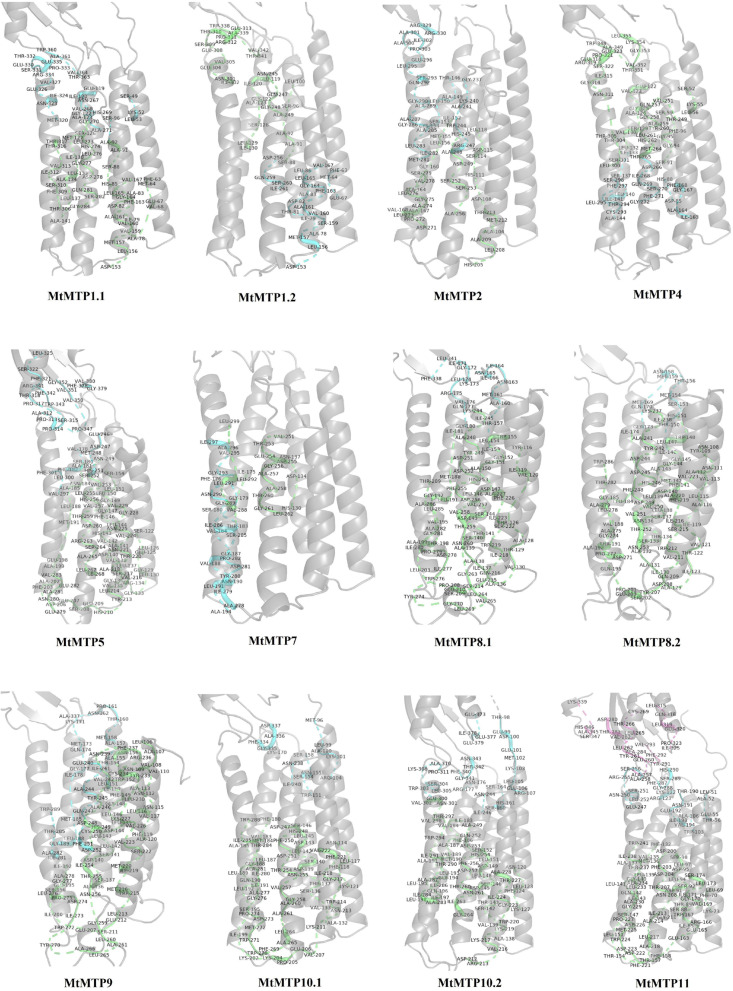
Docking analysis of pocket sites of MTP proteins in *M. truncatula*.

**FIGURE 6:**
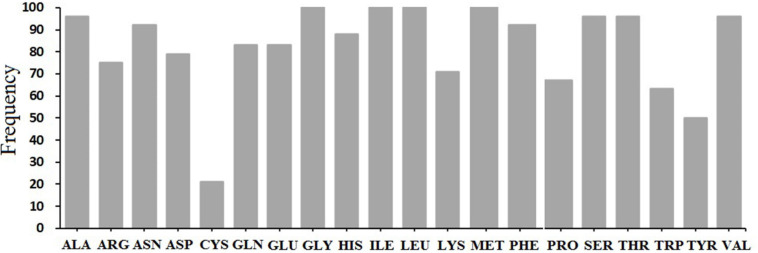
Distribution of ligand-binding sites in all studied MTP proteins.

Based on the results, different pockets were observed, and the key amino acids involved in the function of MTP proteins were predicted (Figure 5). The GLY, ILE, LEU, and MET amino acids were predicted to be the binding residues in the ligand-binding site of all candidate MTP proteins (Figure 6). Followed by ALA, SER, THR, and VAL, the frequency of amino acid residues present in each pocket site was 96% (Figure 6). Overall, GLY, ILE, LEU, and MET are recognized as the key residues in the predicted pocket sites in MTP proteins. These results suggest the importance of these residues in these positions on the DNA molecule and finally, the cellular functional performance.

### Gene Ontology

**FIGURE 7:**
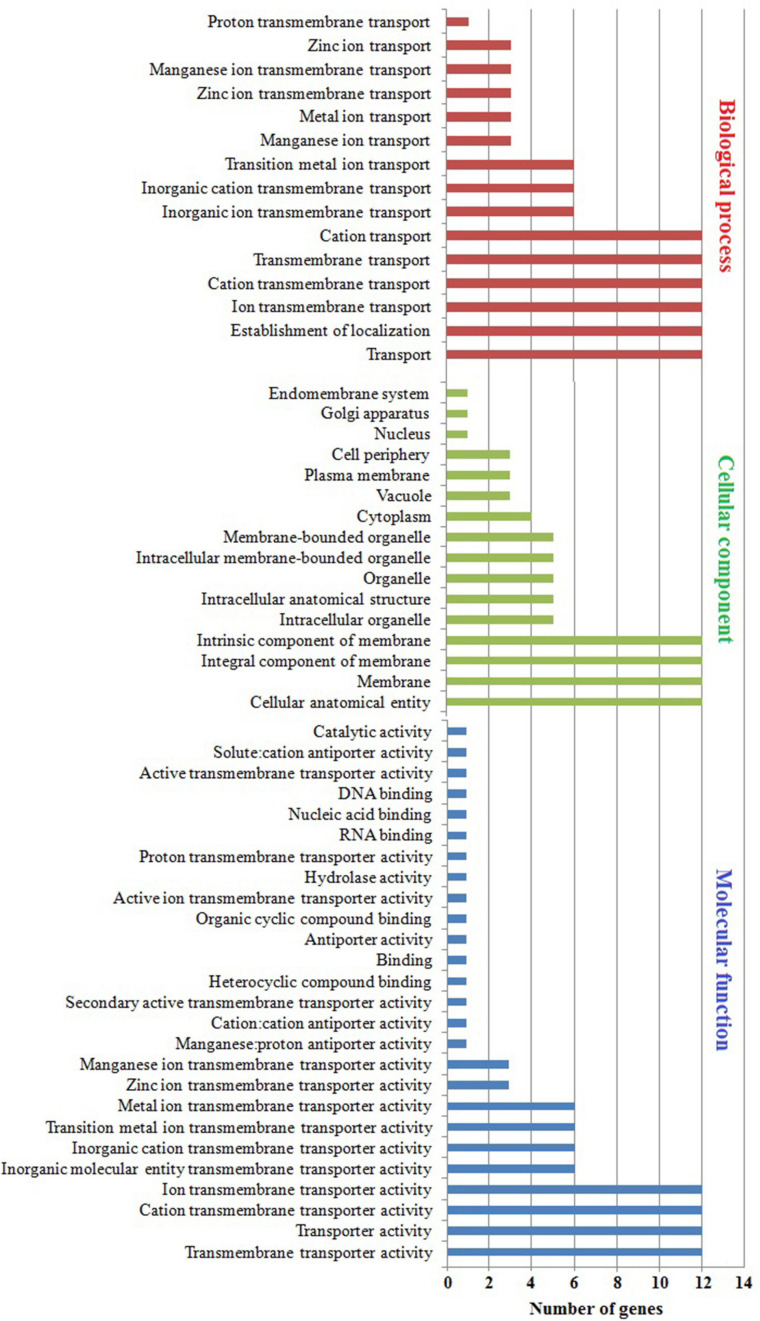
Gene ontology analysis of *M. truncatula MTP* genes. Gene ontology showed the distribution of every *MtMTP* gene in the plant, where a red-colored column shows the cellular component. Conversely, the biological processes in which the *MTP* family participate are shown by a blue-colored column, and the molecular function is shown by a mauve color.

The subcellular localization, molecular function, and biological process were predicted using GO enrichment analysis (Figure 7 and Supplementary Table 5). In subcellular localization analysis, the predicted distribution scores of MTP proteins were as follows: 12/60% in all membranes, 3/15% in the plasma membrane and vacuole, and 1/5% in the Golgi apparatus and root hair. Noticeably, the *MtMTP1.2* gene was localized in 12 subcellular compartments out of all 14, which underlined the significant role of *MtMTP1.2* in metal stress resistance. The collective scores of MTP protein molecules during biological processes were as follows: transmembrane transport of Zn^+^ and Mn^+^ ions was 3/43%, whereas transmembrane transport of cations was 1/14%. More precisely, *MtMTP1.1*, *MtMTP1.2*, and *MtMTP4* play a key role in transmembrane transport of Zn^2+^, whereas *MtMTP8.1*, *MtMTP8.2*, and *MtMTP11* play a crucial role in transmembrane transport of Mn^2+^. Molecular function analysis revealed the significant roles of *MtMTP8.2* and *MtMTP11* in heavy metal processes.

### Gene Expression Analysis by RNA-seq Data

**FIGURE 8:**
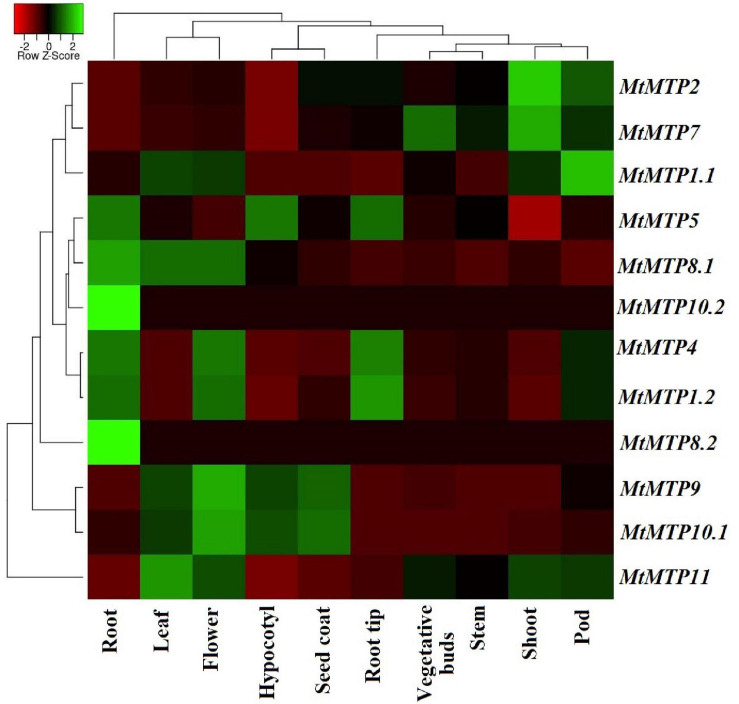
The heat map of 12 *M. truncatula MTP* gene expression profiles based on RNA-seq data. The previous expression has been shown in the root, leaf, flower, hypocotyl, seed coat, root tip, vegetative bud, stem, shoot, and pod tissues.

A heatmap diagram was constructed to show the differential expression level of each *MtMTP* gene in all tissues (Figure 8 and Supplementary Table 6). Comparatively, *MtMTP1.1* displayed the highest expression level in a pod, whereas *MtMTP1.2* displayed the highest expression level in the root tip. Similarly, the highest expression level of *MtMTP2* was observed in plant shoots, whereas the expression level of *MtMTP5* was mild in the hypocotyl, root, and root tip. The expression level of *MtMTP7* was also mild in the buds and higher in the shoot. *MtMTP11* displayed the highest expression level in approximately all tissues, *MtMTP10.1* only in the hypocotyl and seed coat, *MtMTP8.1* only in the root, and *MtMTP9* only in the flower tissue. Noticeably, *MtMTP8.2* and *MtMTP10.2* displayed the lowest expression level in all tissues and the highest expression in the roots.

### Expression Analysis of *MtMTPs* in Response to Heavy Metals Treatment

**FIGURE 9:**
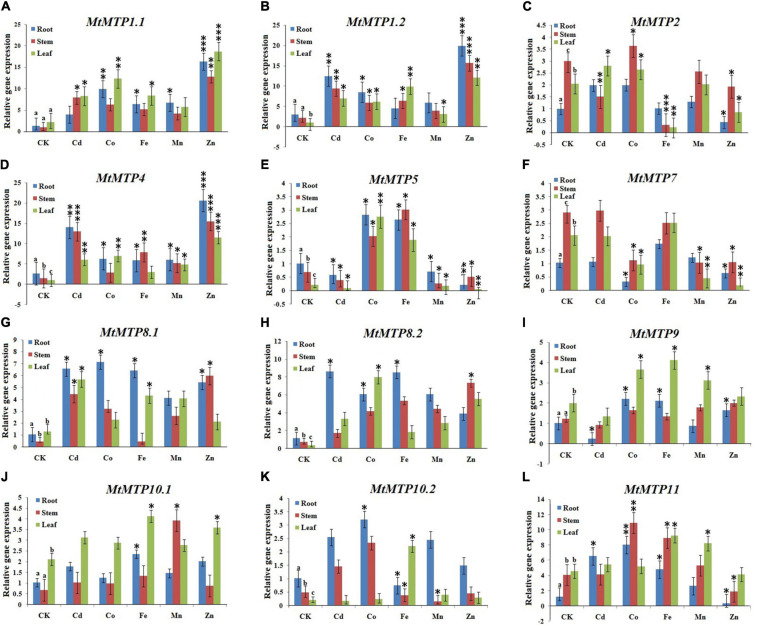
The qRT-PCR expression of the *M. truncatula MTP* genes from root, stem and leaf samples where the genes where showed alphabetically from **(A–L)**. The reactions were normalized using the β-*actin* reference gene. The standard deviations have been represented by the error bars from three independent technical replicates. The mean expression levels of the three replicates were analyzed with the five heavy metal treatments (Cd^2+^, Co^2+^, Fe^2+^, Mn^2+^, and Zn^2+^) using *t*-tests (*p* < 0.05), where as the CK represents the control samples. Different letters (a, b and c) indicate significant differences between the roots, stems, and leaves under normal conditions. Asterisks indicate significant differences between the treatment samples and the corresponding control samples in the roots, stems, and leaves (*n* = 9, *p* < 0.05, Student’s *t*-test).

In this study, expression patterns of *MtMTPs* were investigated using qPCR under different metals treatment. All *MtMTP* genes displayed different gene expression levels under treatment with different types of heavy metals investigated in the following tissues: root, stem, and leaf (Figure 9). In the roots, *MtMTP1.2* and *MtMTP4* displayed the highest expression level, whereas *MtMTP5*, *MtMTP7*, and *MtMTP9* displayed the lowest expression level under Cd^2+^ treatment. Similarly, *MtMTP1.1* and *MtMTP11* displayed the highest expression level, whereas *MtMTP7* displayed the lowest expression level under Co^2+^ treatment. *MtMTP1.1*, *MtMTP4*, *MtMTP5*, *MtMTP8.1*, *MtMTP8.2*, and *MtMTP11* displayed the highest expression level, whereas *MtMTP10.2* displayed the lowest expression level under Fe^2+^ treatment. *MtMTP1.1* and *MtMTP4* displayed the highest expression level, whereas *MtMTP5* displayed the lowest expression level under the Mn^2+^ treatment. *MtMTP1.1*, *MtMTP1.2*, and *MtMTP4* displayed the highest expression level, whereas *MtMTP2*, *MtMTP5*, *MtMTP7*, and *MtMTP11* displayed the lowest expression level under Zn^2+^ treatment.

In the stem, Cd^2+^ treatment resulted in *MtMTP1.2* and *MtMTP4* upregulation, but downregulated *MtMTP2* and *MtMTP5*. Similarly, Co^2+^ treatment significantly upregulated *MtMTP11* but downregulated *MtMTP7*. Fe^2+^ treatment upregulated *MtMTP4*, *MtMTP5*, and *MtMTP11* but downregulated *MtMTP2* and *MtMTP10.2*. Mn^2+^ treatment upregulated *MtMTP4* and *MtMTP10.1* but downregulated *MtMTP5*, *MtMTP7*, and *MtMTP10.2*. Finally, Zn^2+^ upregulated *MtMTP1.1*, *MtMTP1.2*, and *MtMTP* but downregulated *MtMTP5*, *MtMTP7*, and *MtMTP11*.

In the leaf, the expression level of *MtMTP4* was increased in response to Cd^2+^ treatment while *MtMTP5* was downregulated. Similarly, Co^2+^ treatment upregulated *MtMTP1.1*, *MtMTP4*, and *MtMTP5* but downregulated *MtMTP7*. Fe^2+^ treatment upregulated *MtMTP1.2* but downregulated *MtMTP2*. Furthermore, expression levels of *MtMTP1.1* and *MtMTP4* were increased in response to Mn^2+^ treatment, while *MtMTP1.2*, *MtMTP5*, and *MtMTP7* were downregulated. Finally, Zn^2+^ treatment upregulated *MtMTP1.1*, *MtMTP1.2*, and *MtMTP4* but downregulated *MtMTP2*, *MtMTP5*, and *MtMTP7*.

## Discussion

Heavy metals have the biggest impact on the ecosystem and make it unfit for human consumption (El-Sappah et al., 2012; Chidambaram, 2015). Once released into the environment, they accumulate in plants and then in other living tissues via the food chain and cause toxicity even at lower concentrations (Samuel et al., 2020). *MTP* genes (membrane divalent cation transporters) are essential for transporting various heavy metals and enhancing plant tolerance against heavy metal stress (Ricachenevsky et al., 2013). They also have an expected role in plant mineral nutrition maintenance (Liu et al., 2019). Moreover, these metal-binding proteins are now being used as bioenvironmental markers for predicting heavy metal contamination based on their expression levels (Samuel et al., 2021). The *MTP* family has previously been studied in several plants, including *Arabidopsis* (van der Zaal et al., 1999), tobacco (Liu et al., 2019), wheat (Vatansever et al., 2017), and black poplar (Gao et al., 2020), whereas this is the first genomic identification study of the *MTP* family in *M. truncatula*. A total of 12 putative *MTP* genes in *M. truncatula* have been identified, and they were named based on the sequence similarities and orthologous relationships between them and *AtMTPs*.

The broad range of basic physicochemical properties of the *MtMTP* gene including CDS length, protein size, MW, pI, GRAVY, and subcellular localization, was analyzed and was found to be consistent with previous studies indicating huge probabilities of amino acid in metal tolerance (Vatansever et al., 2017; Büyükköroǧlu et al., 2018). MtMTP proteins showed diversity in terms of physicochemical properties, indicating that these gene family members are involved in various pathways and biochemical networks (Ahmadizadeh et al., 2020; Musavizadeh et al., 2021). Consistent with previous studies (Vatansever et al., 2017), our genes are localized to the tonoplast (the vacuole membrane), whereas some might also be localized in the cytoplasm or chloroplast, suggesting that MtMTPs might function as vacuole-localized cation transporters. The phylogeny of MTP proteins between *M. truncatula* and the other five studies was compared (Figure 1). The phylogeny results were aligned to that of the previous studies conducted in various plant species. Multiple homologous pairs were observed in *M. truncatula*, whereas no such pairs were observed in *Arabidopsis*, showing that the *MtMTP* gene family might have undergone gene expansion and/or gene loss in its evolutionary history, probably due to polyploidization events.

About four, two, and six *MtMTP* genes were grouped into Zn-CDFs, Zn/Fe-CDFs, and Mn-CDFs, respectively (Figure 2), considering the implications of phylogenetic distributions in inferring structure and functional roles across species (Eroglu et al., 2017). The various groups are involved in specific mechanisms, and this information could provide clues to predict their function in different species.

In our study, to obtain more knowledge about the gene annotation and expansion mechanism of the MTP gene family in *M. truncatula*, gene synteny and duplication analysis were investigated (Figure 2 and Supplementary Table 2). Two or more genes on the same chromosome are often related to tandem duplication, whereas segmental duplication often occurs in different chromosomes (Schlueter et al., 2007). Our study did not show any of the tandem duplication pairs, whereas 30 segmental duplications occurred, which included *MtMTP2*/*MtMTP8.1*, *MtMTP2*/*MtMTP10.2*, and *MtMTP8.2*/*MtMTP9*. During the evolution process of a plant gene, family duplication events occur, followed by divergence considering standard features that are more related to secondary plant metabolism genes (Ober, 2005).

Almost all subfamilies contained the same numbers of introns and motif sequences that are consistent with the previous studies wherein a similar gene structure was found within the same subfamilies (Liu et al., 2018). For example, all of the gene members of subfamily A contained five introns. However, subfamily F members did not contain any introns. These outcomes indicated that during the *M. truncatula* evolution events of *MtMTPs*, some intron gain and loss occurred. Some genes have no introns but have one exon causing the lower ability of exons in the gain/loss rate due to higher selection pressure in the exon sequences (Harrow et al., 2006). Thus, with all these observations, it is probable that the placement divergences in intron number consider shared events related to the gene family evolution (Jeffares et al., 2006; Rogozin et al., 2012; Babicki et al., 2016).

Protein structures are precisely associated with the functions of genes and can reflect the phylogenetic relationships among them (Zhang et al., 2016; Faraji et al., 2020). The protein structures of all the candidate MTP proteins were modeled at >90% confidence, and their potential active ligand-binding sites were also identified. Some diversity in these protein structures may reflect their different roles in the transmembrane transport process in response to multiple environments. Based on the three-dimensional structure analysis, it can be mentioned that these proteins from multiple clades belong to functionally diverse groups but share a common catalytic mechanism in the stabilization of membrane potential and cellular metal ion homeostasis under stresses, supporting the fact that MTP proteins can play an essential role in intracellular signaling pathways in response to unfavorable conditions.

The GLY, ILE, LEU, MET, ALA, SER, THR, VAL, ASN, and PHE amino acids were predicted as the binding residues in the ligand-binding site of nearly all candidate MTP proteins, which may manifest the importance of these residues in those positions on the DNA molecule and their cellular functional performance. For instance, SER, VAL, and LEU have been identified as the key amino acids involved in regulating responses to stress (Galili and Höfgen, 2002; Beauregard and Hefford, 2006). CREs are non-coding DNA binding by transcription factors and/or other regulatory molecules for regulating the transcription of neighboring genes (Wittkopp and Kalay, 2012). Furthermore, CREs play an essential role in plant response to environmental cues (Yamaguchi-Shinozaki and Shinozaki, 2005), and many of those have been found in the *MtMTP* genes in this study (Supplementary Figure 1). The CRE analysis in the promoter regions of the *MtMTP* genes showed the potential regulatory mechanisms controlling their expression.

The ABRE element is important in ABA signaling and plant response to drought and salt stress in Arabidopsis (Narusaka et al., 2003), and it is the most abundant element present in *MtMTPs*. The SA signaling pathway is mediated by the TCA element that is found in four of the MtMTPs and is responsible for different stress reduction (Mou et al., 2013; Ali et al., 2015). The ARE element was found to be both necessary and sufficient for anaerobic induction of genes (Ali et al., 2021) and as part of the 10 *MtMTP* genes. MBS was found in five of the studied *MtMTPs* in *M. truncatula* and was reported to bind to MYB transcriptional factors involved in drought stress signaling (Shukla et al., 2015). Five of *MtMTP* promoters harbor LTR and are important to induce cold-regulated genes (Brown et al., 2001). Furthermore, only two genes *MtMTP8.1* and *MtMTP10.1*, harbor a TC-rich repeat where they are involved in defense and stress responsiveness (Sazegari et al., 2015). The CGTCA motif is responsible for methyl jasmonate-responsive elements and is important in stress induction (Ali et al., 2018). The detection of these stress-responsive *cis*-elements indicates that the proteins encoding the *MtMTP* genes may have a functional role in heavy metal stress tolerance in *M. truncatula*.

In a detailed evaluation of the MTP proteins, their 3D configuration was predicted, which was considered to be a supportive tool for inspecting their function (Büyükköroǧlu et al., 2018). The four temples in *M. truncatula* MTP proteins indicated that these proteins with heavy metal, where these transport proteins are in plants, are classified into metal-uptake proteins that transport essential and toxic heavy metals to the cytoplasm and metal-uptake proteins. Simultaneously, the other is metal-efflux proteins that help the cell remove any excess heavy metals (Mani and Sankaranarayanan, 2018). Conversely, protein–protein interaction analysis provided us with more knowledge about the plant developmental processes with their interactions with the environment (Struk et al., 2019).

In contrast, gene ontology is a fundamental analysis to predict putative functional contributions across living organisms (Consortium, 2010). Moreover, gene ontology classes and concepts have been used to define the relationships and gene functions existing between these concepts (Purwantini et al., 2014). Our gene ontology analysis has revealed the significant role of the *M. truncatula MTP* genes with heavy metals (Figure 5 and Supplementary Table 5). Furthermore, GO showed the molecular functions of the MtMTPs, where most of them participate in metal-related processes, including transmembrane transporter activity, cation transmembrane transporter activity, transporter activity, and ion transmembrane transporter activity.

Transcriptomic analysis is a proper tool for detecting the existence, structure, and quantity of the RNA in any abiological sample under certain conditions (Zambounis et al., 2020). Thus, the expression profile of all members of the *MTP* gene family from previously published RNA-sequencing data has been investigated, which showed the expression of all gene members in all selected *M. truncatula* tissues (Figure 6 and Supplementary Table 6). Digital data analysis showed that the significant roles of the *MTP* gene could contribute significantly to growth and development. Noteworthy evidence has been obtained about the essential roles of *M. truncatula MTPs* after tissue expression evaluation. For instance, the exclusive expression of the two genes *MtMTP2* and *MtMTP7* was in the root tip more than in any other root part, whereas *MtMTP11* was most abundant in the vegetative buds, indicating that they might be involved in the early root and bud development. Besides the expected vital roles of *MtMTP11* in pod maturation and development, its expression has increased. However, only *MtMTP8.2* and *MtMTP10.2* were weakly expressed in all examined tissues from all *MtMTPs*.

The documented downregulation in some gene expressions is essential for maintaining gene duplicates and ancestral functions (Qian et al., 2010). Hence in our study, the downregulation of *MtMTP8.2* and *MtMTP10.2* expression is expected to be vital to keep their biological functions and to maintain them from losing them during cell evaluation. The reliability of the transcriptome data was further validated using quantitative reverse transcription-polymerase chain reaction (qRT-PCR) (Figure 7); however, the minor asymmetry between both analyses may be due to different growth conditions and *M. truncatula* varieties, which finally affected the spatial expression. The expression behavior of *MTP* genes was examined under five divalent metals (Mn^2+^, Cd^2+^, Co^2+^, Fe^2+^, and Zn^2+^). Numerous studies in other plants indicated the significant roles of the *MTP* gene family to enhance plant tolerance against these metals (Montanini et al., 2007; Gao et al., 2020) as it was found that metal-efflux transporters from the cytoplasm mainly transport Zn^2+^ but also transport Ni^+2^, Co^2+^, Cd^2+^, Fe^2+^, and Mn^2+^ (Ricachenevsky et al., 2013).

The transcript accumulation transcription of *MTPs* in response to various heavy metals is varied and complicated, although the gene expression response to different stresses is usually reflected in corresponding gene roles. In *Arabidopsis*, the tonoplast-localized Zn transporter *AtMTP1* showed slight changes in expression with excess Zn exposure at both transcription and translation levels (Dräger et al., 2004; Kobae et al., 2004). Moreover, despite the high expression of the *CsMTP1* encoded protein, the gene expression was steady under the high concentration of Zn^2+^ in cucumber (Migocka et al., 2015).

As mentioned before, the *AtMTP12* upregulation was not due to Zn^+^ treatment but due to the formation of a heterodimeric complex with *AtMTP5* for Zn^+^ transport (Fujiwara et al., 2015), same in tobacco (Liu et al., 2019). Moreover, variable concentrations of Mn^2+^ slightly affect the expression of Mn-CDFs (*AtMTP8*, *AtMTP9*, *AtMTP10*, and *AtMTP11*) (Delhaize et al., 2007), same with tobacco (Liu et al., 2019). Contrarily, all Zn-CDF members displayed significant variation in their expression under Zn^2+^ treatment, such that *MtMTP2* and *MtMTP7* of Zn/Fe-CDFs were downregulated under treatment of a higher concentration of Zn^2+^. Furthermore, *MtMTP10.1* of the Mn-CDF class was highly affected by Mn^2+^ accumulation only in the stem. Our findings provide deep insights in the molecular function of the *MTP* genes in *M. truncatula* under various heavy metal stresses, which will invite researchers to precisely identify the function of the desired *MTP* genes in *M. truncatula via* wet lab experiments.

Generally, these results would provide essential clues in clarifying the roles of *MtMTPs* in heavy metal tolerance and the mechanism of heavy metal transport mediated by MtMTP proteins. Altogether, these results would lay a theoretical and practical foundation for the functional characterization of *MtMTP* genes in future studies. Furthermore, the highest expressed *MTPs* (*MtMTP1.1*, *MtMTP1.2*, and *MtMTP4*) can be used as bioenvironmental markers for predicting heavy metal contamination based on their expression levels.

## Conclusion

The first genome-wide study of the *MTP* gene family in *M. truncatula* has been provided, providing important comparative data for evolutionary relationships. Twelve identified *MTP* genes were phylogenetically divided into three major substrate-specific clusters (Zn/Fe-CDFs, Mn-CDFs, and Zn-CDFs), and seven groups seemed to have undergone expansion and gene loss after polyploidization through segmental duplication. The *MtMTP* transcripts under plant hormones and heavy metal stresses showed that they might participate in the plant response signaling pathway, which was related to the wide distribution of the CREs in their upstream regions. GLY, ILE, LEU, MET, ALA, SER, THR, VAL, ASN, and PHE amino acids were predicted to be the binding residues in the ligand-binding site of all these proteins. The expression patterns of *MtMTP* members in response to various heavy metals at different tissues indicated the significant role of these genes in the growth and development of *M. truncatula*. Furthermore, our gene expression analysis of various heavy metals has revealed the significant roles of the *MTPs*, particularly those of *MtMTP1.1*, *MtMTP1.2*, and *MtMTP4*, in plant tolerance to heavy metal stresses.

## Data Availability Statement

The original contributions presented in the study are included in the article/Supplementary Material, further inquiries can be directed to the corresponding author/s.

## Author Contributions

AE-S, JL, ZX, and KE-T: conceptualization. AE-S and RE: writing original draft. AE-S, RE, KE-T, and JL: bioinformatics and formal analysis. AE-S: drawing figures. AE-S, JL, and RE: experiment design and perform. AE-S, AE, KE-T, YW, QH, KE, YZ, and MA: editing. AE-S, YZ, JL, QY, and MA: review. AE-S, KE-T, and RE: writing final manuscript. All authors critically revised the manuscript and approved the final version.

## Conflict of Interest

The authors declare that the research was conducted in the absence of any commercial or financial relationships that could be construed as a potential conflict of interest.

## Publisher’s Note

All claims expressed in this article are solely those of the authors and do not necessarily represent those of their affiliated organizations, or those of the publisher, the editors and the reviewers. Any product that may be evaluated in this article, or claim that may be made by its manufacturer, is not guaranteed or endorsed by the publisher.

## Footnotes

1
http://www.medicagogenome.org/

2
https://www.sanger.ac.uk/tool/pfam/

3
https://blast.ncbi.nlm.nih.gov/Blast.cgi

4
https://phytozome.jgi.doe.gov/

5
http://smart.embl-heidelberg.de/

6
http://www.expasy.org/tools/protparam.html

7
https://wolfpsort.hgc.jp/

8
http://cucurbitgenomics.org/

9
http://plantgdb.org/PtGDB/

10
https://rapdb.dna.affrc.go.jp/

11
https://www.wheatgenome.org/

12
https://www.wur.nl/en/show/Mapchart.htm

13
http://www.ebi.ac.uk/Tools/psa/

14
http://circos.ca/

15
http://gsds.cbi.pku.edu.cn/

16
http://meme.nbcr.net/meme3/meme.html

17
https://www.dna.affrc.go.jp/PLACE/?action=newplace

18
http://bioinformatics.psb.ugent.be/webtools/plantcare/html/

19
http://www.sbg.bio.ic.ac.uk/phyre2/html/page.cgi?id=index

20
https://proteins.plus/

21
https://pymol.org/2/

22
https://www.blast2go.com

23
https://mtgea.noble.org/v3/

24
http://heatmapper.ca/
