# Supplementary material for: Genome-Wide Identification and Expression Analysis of Metal Tolerance Protein Gene Family in Medicago truncatula Under a Broad Range of Heavy Metal Stress
Source: Front Genet. 2021 Sep 7;12:713224. doi: 10.3389/fgene.2021.713224 (PMC8482800; doi:10.3389/fgene.2021.713224)
Supplement: Supplementary file 1 [file Table_1.DOCX]

**
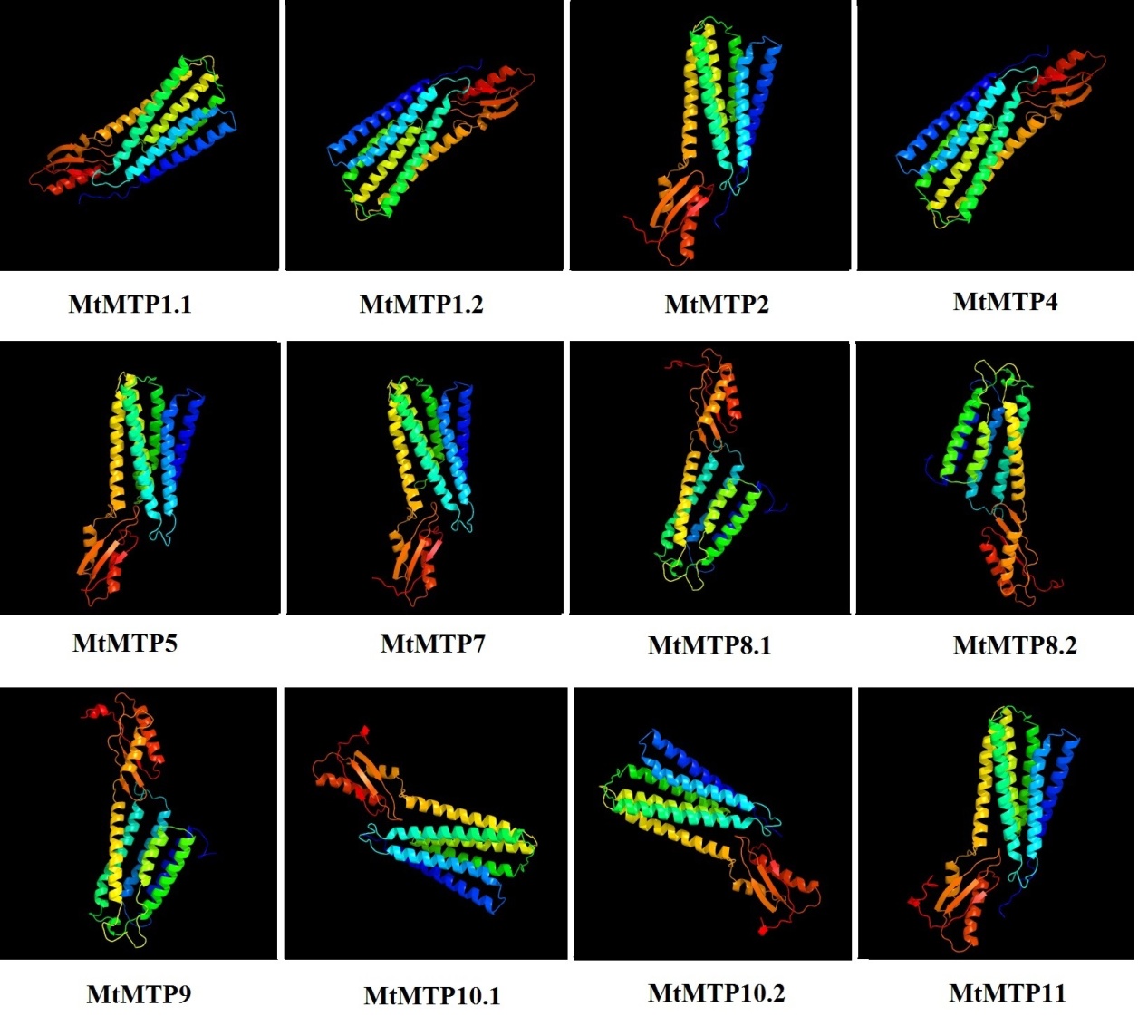
**

**Figure S1. Predicted 3D models of *M. truncatula* MtMTP proteins**. Models have been generated by using the Phyre 2 server in intensive mode. Models were visualized by rainbow colour from N to C terminus.
