# Supplementary figures and images for: Genome-Wide Identification and Expression Analysis of Metal Tolerance Protein Gene Family in Medicago truncatula Under a Broad Range of Heavy Metal Stress
Source: Front Genet. 2021 Sep 7;12:713224. doi: 10.3389/fgene.2021.713224 (PMC8482800; doi:10.3389/fgene.2021.713224)

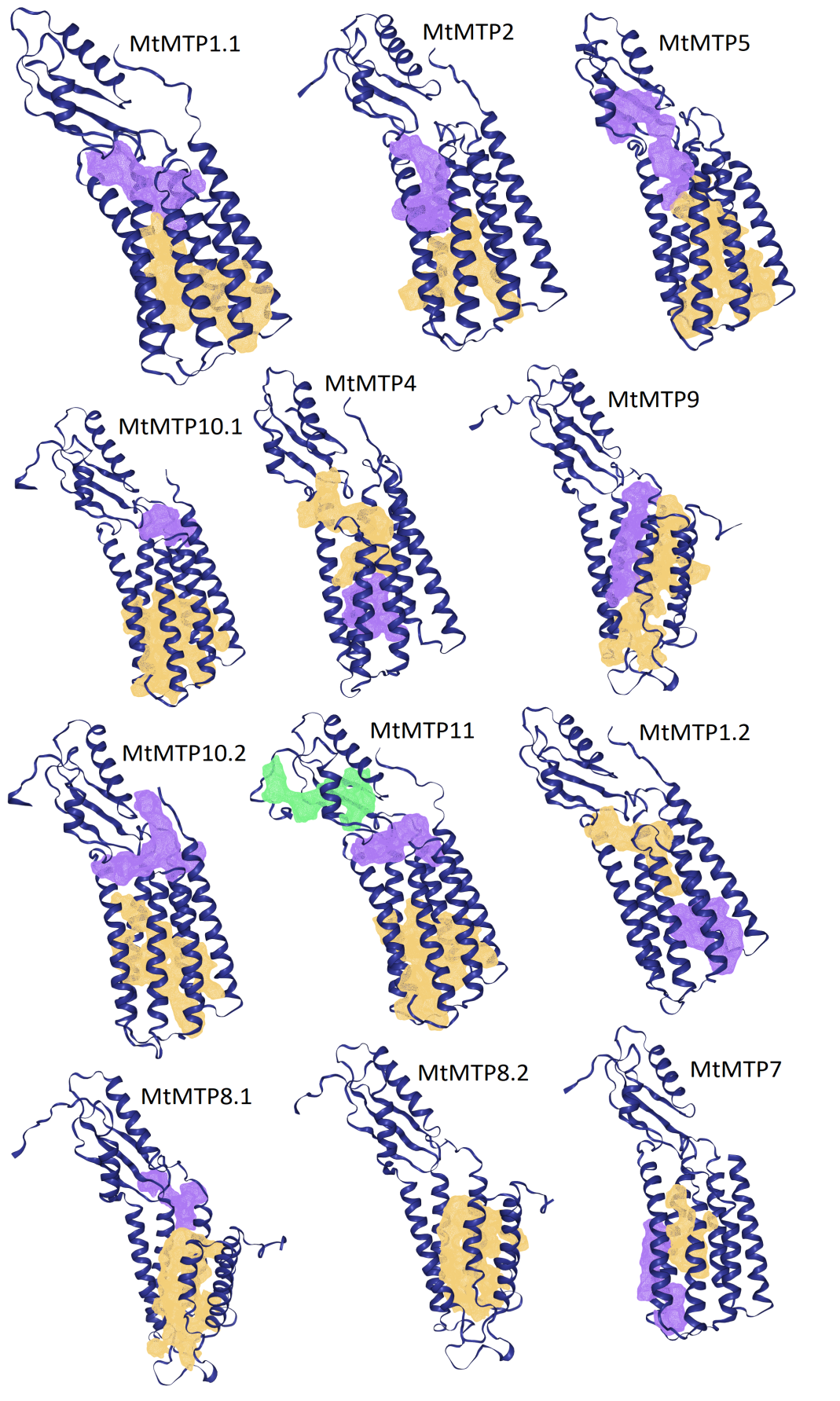


**Figure S2. The protein channel regions predicted in MTP proteins in *M. truncatula***

Supplement: Supplementary file 2 [file Table_2.DOCX]
