# Supplementary material for: Genome-Wide Identification and Expression Analysis of Metal Tolerance Protein Gene Family in Medicago truncatula Under a Broad Range of Heavy Metal Stress
Source: Front Genet. 2021 Sep 7;12:713224. doi: 10.3389/fgene.2021.713224 (PMC8482800; doi:10.3389/fgene.2021.713224)
Supplement: Supplementary file 3 [file Table_3.DOCX]

| No | Gene ID | Primers sequences | **Tm** |
| --- | --- | --- | --- |
| 1 | [**LOC11443599**](https://www.ncbi.nlm.nih.gov/gene/11443599) | **Forward** ACCCACACTCCGAATAAACCC  **Reverse** GTCCATGGTGAGCGCTTTGT | 60.5 |
| 2 | [**LOC25500609**](https://www.ncbi.nlm.nih.gov/gene/25500609) | **Forward** TAGCCGCTCAGAACAAACCT  **Reverse** TGAGAGCTGTGCGCATCCA | 61.1 |
| 3 | [**LOC25484765**](https://www.ncbi.nlm.nih.gov/gene/25484765) | **Forward** TCCCCTTGCTGGACTTCTTGTC  **Reverse** AGTTGAACCAGCTCTCCTTCCT | 61.5 |
| 4 | [**LOC25492544**](https://www.ncbi.nlm.nih.gov/gene/25492544) | **Forward** TGTCTTCTGGGTTCTACACGTTG  **Reverse** AGGCGCTTTCTCGTGTTCCA | 61 |
| 5 | [**LOC11428206**](https://www.ncbi.nlm.nih.gov/gene/11428206) | **Forward** AATGGCACCTCCCAGCATCC  **Reverse** AACCACATGACCAGGCACCA | 62.2 |
| 6 | [**LOC25491241**](https://www.ncbi.nlm.nih.gov/gene/25491241) | **Forward** AGTTGTGCATTCGGTTGCCG  **Reverse** ATAGCATCTGGTGCACGCCT | 62 |
| 7 | [**LOC11413755**](https://www.ncbi.nlm.nih.gov/gene/11413755) | **Forward** TCTCCAACGCCACCGCTTTA  **Reverse** CGGTCAGATTCCACCACAGC | 61.5 |
| 8 | [**LOC11425928**](https://www.ncbi.nlm.nih.gov/gene/11425928) | **Forward** TCCTCATTTCTCCACATCCACCA  **Reverse** GAGCCCGCTTCTCCATGTCTT | 61 |
| 9 | [**LOC25501161**](https://www.ncbi.nlm.nih.gov/gene/25501161) | **Forward** ACCGTCAACCACGTTCCTTCA  **Reverse** TGCTACCTTGCGTTGCTTCT | 61 |
| 10 | [**LOC25486917**](https://www.ncbi.nlm.nih.gov/gene/25486917) | **Forward** AACAACGCAAGGTAGCCGAG  **Reverse** GTACTGCCGTCCTCTCGCTC | 61 |
| 11 | [**LOC11432698**](https://www.ncbi.nlm.nih.gov/gene/11432698) | **Forward** TGGTGGCTTGACCCTCTTGG  **Reverse** AGGTACTCCGGTGGAGCTGT | 62 |
| 12 | [**LOC11438849**](https://www.ncbi.nlm.nih.gov/gene/11438849) | **Forward** CGGTCATGGCGGTTGAACTT  **Reverse** CAAGCACTTCAACCTGCTGCT | 61.4 |

Table S1. The real-time PCR primers of 12 *MTP* gene family
