# Supplementary material for: Genome-Wide Identification and Expression Analysis of Metal Tolerance Protein Gene Family in Medicago truncatula Under a Broad Range of Heavy Metal Stress
Source: Front Genet. 2021 Sep 7;12:713224. doi: 10.3389/fgene.2021.713224 (PMC8482800; doi:10.3389/fgene.2021.713224)
Supplement: Supplementary file 5 [file Table_5.DOCX]

**Table S 3. Analysis of the 10 conserved motifs of MtMTP genes in *M. truncatula*.**

| **Motif** | **Width** | **Best possible match** |
| --- | --- | --- |
| 1 | 50 | CWTTGGAYTCTCTNYTGGAYCTSTTGKCYGGDTKYATAHTKTGGTTCACT |
| 2 | 50 | CCDGTDGGWATHMTTGTTTTTGCWBCYGTBATGGCAACHBTTGGHTTBCA |
| 3 | 50 | GCYKATGCWSMRSATYHBHTYTYYGATGTKRYWACWAATGCVRTYGBWYT |
| 4 | 50 | ACWGTDHGHGCWTACACATTTGGTKCDCADTAYTTTGTTGADGTTGACAT |
| 5 | 50 | CAARARAARCTHGARVWWYTKYCTGAADTTGARAGWGCTTTTGTTCATHT |
| 6 | 50 | GSTGCTNTBYTWGCTRWBAARTTNDWHTGGTGGMTKGMTCCDWTTGGWGC |
| 7 | 50 | ATGARNAMMCYAAAYMNNTATCAVTAYCCTATTGGAAAGMWRMGKATGCA |
| 8 | 50 | ACDGTGATGGARAATGYAKNKTCAYTNRTBGGAMRAWCWGCWCCWCCTGA |
| 9 | 50 | YTDYCAGMDGAHWTGHCWBTRAAHSAAGCACATRACATTGGDGARACWCT |
| 10 | 41 | TGYKKCMATCRDGASYRGATCHKTRGYBRTGRYTGYRTCMA |
